# Refugees from countries with complex political contexts: politically-informed approach to health and mental health services

**DOI:** 10.3389/fpubh.2023.1323259

**Published:** 2023-12-20

**Authors:** Elena Cherepanov

**Affiliations:** Independent Consultant in Global Mental Health, Boston, MA, United States

**Keywords:** complex political context (CPC), global mental health, Mr. Jones Dilemma, political adversity dimension in care, refugees, social cognitions, survival messages, trauma aggregation

## Introduction: time to talk politics?

The studies of the impact of refugee resettlement have demonstrated that refugees enrich the social, cultural, and economic life in host communities (1). This transition is not always easy: during the integration process, refugees must overcome cultural and language barriers and cope with trauma, losses, and adjustment issues. Refugee health and mental health (MH) care and psychosocial support providers (in the future, “Providers”) stride to address the barriers to accessing resources amid the sensitive political realities of current migration trends.

Culturally responsive trauma-informed care in refugee resettlement emphasizes the importance of cultural sensitivity, contextual relevance, and situational appropriateness in service provision (2). Incorporating such practices in the health and human services supporting refugee resettlement allows addressing vulnerability while promoting resilience. Cosgrove et al. noted that in the current climate of global geopolitical instability and increased violence, political experience has become a prominent factor affecting refugees' health and MH wellbeing (3). The authors suggest that politically informed approaches broaden the perspective on Global Mental Health, away from the legacy of the over-pathologizing trend maintained by Western MH.

The serious and often intergenerational impact of tyranny, genocide, or politically-motivated violence on refugees' mental health has been noted in various populations: Holocaust survivors (4), Asian American families (5, 6), immigrants Latinos (7), or refugees from the post-Soviet countries (8). Although studies spotlight the various facets of such experience, the multitude of evidence points to the importance of recognizing its impact.

This paper draws attention to the historical-political dimension of the refugee experience and advocates for increased political competencies in refugee care. It would mean that Providers are aware of the geopolitical situation in different parts of the world and routinely consider the refugees' exposure to political adversity as a factor potentially affecting the therapeutic alliance and access to care.

This need becomes particularly apparent when serving refugees who fled countries with complex political contexts or CPCs. The paper discusses the practical implications of previous findings about the MH impact of social and political victimization in the health and MH services provision. It draws on the literature review and the author's Global Mental Health (GMH) experience in providing refugee services (8, 9).

## Complex political context

Cherepanov defines *Complex Political Context (CPC)* as settings affected by complex political dynamics, social instability, political or sectarian violence, and ethnic cleansing, resulting in gross violation of human rights and massive trauma (9).

Authoritarianism, dictatorial, and nationalistic regimes unleash violence against political, religious, ethnic, or gender-identity minority groups. These groups become a target of political violence, institutional discrimination, and systematic persecution and are subjected to police brutality and torture. WHO defines political violence as using power and force to achieve political goals (10). This definition also includes deprivation, the deliberate denial of basic needs, and human rights.

Widespread human rights abuses and politically motivated violence distinguish CPCs from other settings with high criminal violence rates.

Authoritarian leaders abuse political power to exercise power and control. They employ coercion, terror, threats, torture (hard tactics), indoctrination, and brainwashing (soft tactics) to intimidate and control the citizens. In states with CPCs, the administration deliberately fosters divisiveness and inflames racial and ethnic tensions by blaming the victims (11).

The experience of politically motivated victimization and abuse carries a profound psychological impact, which can reverberate across generations (4, 5, 8, 12).

### Mental health impact

When refugees flee, they not only carry with them their scarce belongings. Their baggage also includes culture, memories, experiences, and learned social cognitions and beliefs, which can be particularly pervasive when formed as a survival skill.

Political violence affects individual and collective functioning and health (13). Survivors of political persecution often report chronic fear, anxiety, depression, and unpredictability. The trauma of social and political victimization and persecution is complex and chronic: cumulated hardship and multiple and ambiguous losses (e.g., missing, imprisoned, or murdered family members or friends). Many become victims or witness violence, are tortured, or know those who were.

Coping with political adversity may carry similarities with dealing with the complex trauma of interpersonal abuse: Learned social helplessness, social and emotional withdrawal, or increased alcohol consumption and other forms of self-destructiveness are commonly reported coping strategies with societal victimization. Socially avoidant behavior helps keep a low profile, avoiding exposure or unwanted attention. In CPCs any attention from the authorities is unwanted even when this attention is positive (8, 9).

The experience of political persecution tends to create mutual distrust in the community and distrust toward governmental officials and health providers, complicating access to health care and community integration.

### Social cognitions reflect social fears and powerlessness

In refugees fleeing persecution, the experience of targeted and deliberate victimization increases social vulnerability. Even when health and MH services are available, distrust can hinder their access. There is a pervasive belief that health and MH issues may be used as an excuse to thwart the immigration process and deny permanent residency.

Distrust in authorities prevents refugees from seeking help and reporting domestic violence or other crimes to law enforcement (6, 7, 9). It increases refugees' risk of re-victimization and being taken advantage of.

Survival experience solidifies in the form of so-called survival messages (SM), the condensed survival life lessons communicated in the family, and sometimes mistaken for cultural wisdom (14, 15). Analysis of SMs allows us to capture the implicit beliefs and takeaways from the deliberate victimization experience. Cherepanov identified similar life lessons in people coming from different countries affected by CPCs (9):

- No one, especially strangers or officials, can be trusted. Even close friends or family can betray.
- Exposing personal weaknesses, including fears, makes it easier to be taken advantage of. Disclosing MH issues is often perceived as disclosure of vulnerabilities.
- Reaching out and accepting help can create vulnerabilities that adversaries will exploit.

These social beliefs reflect social fears, helplessness, and powerlessness. They are not conducive to sharing trauma experiences or reaching out for help and can add to the barriers to accessing health and MH services in refugees.

### Community support eroded by mutual distrust

Community support is critical in trauma recovery and creating a safe and supportive environment conducive to integration. It is not always the case for refugees affected by CPCs, where communities may lack aggregation, a striking difference from the post-disaster recovery community. The phenomenon of *aggregation* refers to the emergence of shared trauma experience or the emergence of a special mental space where survivors feel safe to disclose and can relate to and validate each other's experiences. Distrust, social fear, shame, and moral injury associated with CPCs' experiences prevent traumatic experiences from aggregating. Lack of aggregation diminishes the community's capacity to provide mutual support (8).

Stresses of resettlement are often credited for strengthening community cohesion and support, but they also can also lead to further social exclusion of already marginalized people. In this way, the communities of refugees often reproduce the social and political division and tensions that existed in their homeland.

For example, LGBTQ minorities who were persecuted at home often continue hiding their identity from the community in the US out of fear of re-victimization (Cherepanov, Personal communication).

Many political refugees are acutely aware that the community may share some beliefs pertinent to the country of origin, and there may be former perpetrators who can threaten their families. In the community, people with troubled histories may want to avoid recognition by any means.

When the community is unwilling or unable to support refugees or further marginalize them, Providers may need to take a more proactive position, scale up their support, and engage the support systems outside the ethnic community.

### Political situation at places of resettlement

Social and political dynamics at the places of resettlement can exacerbate the refugee's traumatic experience.

During the political campaigns of 2016 and 2020 in the US, some refugees shared with me that political rhetoric reminded them of their homeland. They did not feel safe and reported recurrences of traumatic memories and nightmares (Cherepanov, personal communication).

At the places of resettlement, refugees can also encounter anti-immigrant sentiments amid prejudices and real or perceived competition for resources. Hostile community attitudes can increase the sense of uncertainty and fear about the future. They compound the sense of not being welcomed, marginalized, and unsafe, leading to emerging ethnic enclaves with a siege mentality and internal violence where victims are reluctant to report abuse and disclose crimes.

### Attitudes toward providers reflect apprehension toward the authorities

Not surprisingly, the CPC's experience affects trust and rapport with Providers who may be perceived as government officials. And it is not incidental.

In many countries with CPCs, there is a well-documented use of MH services for punishment and surveillance. For example, the government in the Soviet Union and now Russia has been employing punitive psychiatry, involuntarily confining dissidents to the psychiatric facility and forcing on them anti-psychotic medications (16). In the 1970s, Somalia openly used psychologists to report disloyalty to the government. Most recently, Kurpatov, the president of the Professional Medical Psychotherapeutic Association in Russia, proposed an amendment to the professional code of ethics for psychologists and psychiatrists, requiring that they inform officials of their patients who don't support military operations in Ukraine. They are also expected to use MH interventions to motivate young people to join the army and to report draft dodgers (17).

## Recommendations for health communication and interventions

Psychoeducation, integrated primary care, and psychosocial services demonstrated the advantages of addressing the complex needs of refugees in a less stigmatizing and more effective manner (18). In addition, in many cultures, psychological distress presents and communicates in the form of somatic symptoms, while Providers sometimes struggle to explain to patients the benefits of standalone MH services. Primary care providers are well positioned to serve as a foot in the door to identify and engage those needing MH support.

When assisting and treating refugees, the politically-informed Providers:

Curious about the history and current political situation in the refugees' homeland.

Are aware of barriers preventing refugees from accessing services and make efforts to address these barriers. For example, using phone interpreters where a client can remain anonymous even when community or family members are available to translate.

Are mindful of the power differential and the disproportionate weight given to providers' every word.

Are aware that refugees often come from countries where it is not safe to trust law enforcement. Reluctance to disclose victimization makes safety and trauma assessments less reliable.

Are aware that the community may not be able or willing to support some refugees. In these cases, seeking support outside of the community is needed, and Providers can assist with it.

Are aware of the stigma associated with MH and certain physical conditions, and consequent internalized stigmatization. Shifting the focus to less contentious issues like problems with sleep or stress management can help engage refugees in health and MH services.

Balance information gathering during the intake with allowing patients to guide the process: “Tell me what you believe is important to know about you.” This is done to avoid the appearance of interrogation.

Are aware that in most interventions, trust, and telling the truth are desirable but is not required. Instead, offer a person to volunteer the information that may be important for a provider to know.

Are aware that medical procedures can retraumatize some refugees, and MH support may be required. For example, torture survivors' experiences of receiving surgical treatment may be indicative of re-traumatization (19).

### Additional consideration

Perpetrators also may need MH services. In CPCs, the line between perpetrator and victim can be blurry: a perpetrator can become a victim in the next cycle of political violence and vice versa. Addressing their trauma offers the opportunity to break the cycle of violence (8).

## Ethical considerations: dual representation and the Mr. Jones Dilemmas

Servicing refugees exposes MH providers to unique ethical dilemmas (20). One is *the dual representation dilemma*, which highlights the potential conflict between the norms, attitudes, or political convictions adopted by refugees and the society with which Providers identify. This dilemma common in refugee work can become a point of contention when working with refugees who may internalize the beliefs associated with CPCs.

*Advocacy vs. Do No Harm Mr. Jones's Dilemma* underscores the high risks of exposure due to advocacy. This dilemma is depicted in the Mr. Jones film (21) and is named after journalist Gareth Jones, who, in 1933, traveled to the Soviet Union, where he discovered evidence of the Holodomor. This artificially created famine resulted in mass starvation and death in Ukraine. Upon return, Jones had to decide whether to advocate for victims and, in this way, put them in more danger or expose the atrocities. He chose the latter, which is how we know about them now. Similarly, Providers' advocacy may come at a high price for the exposed refugees and their families remaining in the CPCs (20).

## Discussion

Politically informed care is essential in addressing barriers to health and MH care in refugees from countries with CPCs. With the rising political instability and authoritarian and nationalistic tendencies around the world, political violence has become a routine part of the GMH experience. Exposure to political victimization affects many facets of the refugees' wellbeing and integration and becomes yet another barrier to accessing health and MH services.

For a long time, health and MH care professionals have been shying away from discussing the political dimensions of the refugee experience, especially when it interacts with the political developments at the places of resettlement. At this time, Providers can no longer ignore the political facet of adversity. Political competencies must become at least as important as trauma-informed care or cultural sensitivity. Such an approach will allow providers to understand better the individual needs of the people served within a broader societal and political context and develop contextually appropriate strategies for addressing them.

I see the next steps in developing the guidelines on routine screening for politically adverse and intergenerational trauma experiences, conceptualizing politically informed approaches in global mental health and refugee health care, and outlining the best practices.

## Author contributions

EC: Writing – original draft.
